# Persistent Pain After Cardiac Surgery: Prevention and Management

**DOI:** 10.1177/10892532211041320

**Published:** 2021-08-20

**Authors:** James C. Krakowski, Matthew J. Hallman, Alan M. Smeltz

**Affiliations:** 1 University of North Carolina at Chapel Hill, NC, USA

**Keywords:** cardiac surgery, persistent postoperative pain, multimodal analgesia, opioids, postoperative complications

## Abstract

Persistent postoperative pain (PPP) after cardiac surgery is a significant complication that negatively affects patient quality of life and increases health care system burden. However, there are no standards or guidelines to inform how to mitigate these effects. Therefore, in this review, we will discuss strategies to prevent and manage PPP after cardiac surgery. Adequate perioperative analgesia may prove instrumental in the prevention of PPP. Although opioids have historically been the primary analgesic approach to cardiac surgery, an opioid-sparing strategy may prove advantageous in reducing side effects, avoiding secondary hyperalgesia, and decreasing risk of PPP. Implementing a multimodal analgesic plan using alternative medications and regional anesthetic techniques may offer superior efficacy while reducing adverse effects.

## Introduction

Persistent postoperative pain (PPP) is a devastating complication that affects both patients and health care systems. The incidence of moderate to severe PPP can vary widely based on surgery type, from <5% to >80% of cases performed.^1-3^ It can be difficult, however, to compare studies that report the incidence of PPP due to differences in criteria used and time points assessed. According to the International Association for the Study of Pain, PPP is defined as pain that persists beyond 3 months after surgery, can be continuous or intermittent, and is attributable to the surgical insult, excluding other potential etiologies.^4^ Up to 43% of patients suffer PPP at 3 months after cardiac surgery.^1^ A meta-analysis that included 11 057 cardiac surgical patients across 23 studies demonstrated a 37% incidence of PPP in the first 6 months and up to 17% at 2 years after surgery.^5^ A retrospective analysis of 35 817 cardiac surgical patients registered in a national administrative claims database of private payers revealed that nearly 1 in every 10 patients who were previously opioid naïve developed opioid use that persisted >3 months after surgery and associated higher oral morphine equivalents prescribed with an increased risk of persistent opioid use.^6^ Patients with PPP experience decreased health-related quality of life, effectively rendering the surgical intervention a means to displace the focus of their suffering instead of alleviating it. Beyond suffering, pain impedes critical postoperative rehabilitation, resulting in functional disability that prevents return to work and a normal life. On a larger scale, PPP can result in medicolegal actions, disparate economic impact, and increased medical costs and resource utilization.

Of cardiac surgical patients with PPP, half have reported it to be moderate to severe and most have described it as neuropathic in quality, the primary location of pain in the chest, the next being the leg in patients having undergone saphenous vein harvesting.^5^ Risk factors for PPP after cardiac surgery include young age, female gender, preexisting pain, anxiety, a catastrophizing mindset, higher body mass index, and a history of osteoarthritis.^5,7^ Of note, more intense acute postoperative pain has been associated with a greater incidence and severity of PPP.^5,7^

## Prevention of PPP

**Figure 1.**
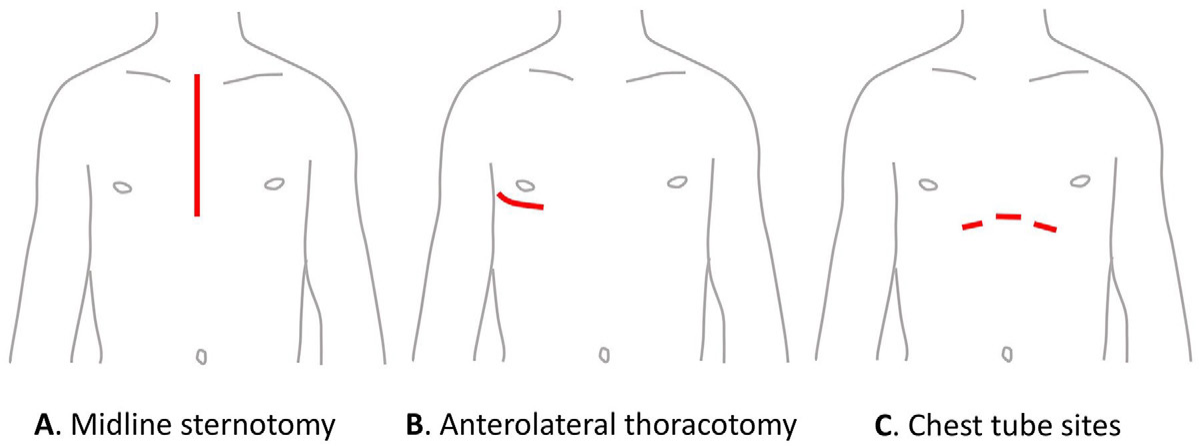
Commonly used incision sites for cardiac surgery.

Because poorly controlled acute pain has been shown to increase the risk of chronic pain and related PPP.^8,9^ Likewise, aggressive preventive analgesia has been associated with decreased PPP.^10^ Therefore, adequate understanding and management of perioperative pain constitutes a fundamental strategy in PPP prevention following cardiac surgery. Risk factors for acute postsurgical pain include young age, longer duration of surgery, and thoracotomy, as compared with surgical access at other sites (see Figure 1).^11^ Direct mechanisms of acute cardiac surgical pain include incision, rib cage retraction-related osteoarticular hypermobilization and rib trauma, tissue dissection, puncture and cutdown for vessel cannulation and harvest, chest tube entry sites and pleural irritation, and muscle spasm from operative positioning and prolonged bed rest. With clear implications for impact on recovery, severe pain has been reported to occur during periods of rest for half of patients and during coughing and deep breathing for three quarters of post-cardiac surgical patients.^12^ Acute pain intensity is typically highest during the initial 24 hours after surgery and abates rapidly throughout the first week.^13^ During this early recovery period, the primary location of pain shifts from the incision site and epigastric region to the shoulders, back, and, for patients having undergone saphenous vein harvesting, the leg. Presumably, as the incision heals and drains are removed, those areas hurt less, which then exposes pain from other sources that had already been present but was previously less intense by comparison.

### Rationale for Opioid-Sparing Techniques

**Figure 2.**
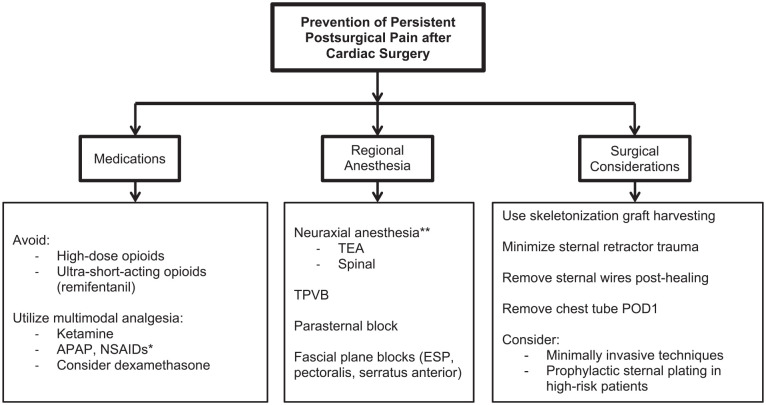
Recommendations summary for the prevention of persistent postsurgical pain after cardiac surgery. Overarching goals include improvement of acute perioperative pain control (prevention of severe acute pain) and reduction in opioid requirements and associated side-effects. In general, a multimodal, opioid-sparing approach via a multidisciplinary team is recommended. *NSAIDs should be used with caution in patients with cardiovascular disease. **Neuraxial anesthesia should be performed with caution in the anticoagulated patient (APAP, acetaminophen; NSAIDs, non-steroidal anti-inflammatories; TEA, thoracic epidural analgesia; TPVB, thoracic paravertebral block; ESP, erector spinae plane block; POD, postoperative day).

Cardiac anesthesia has historically relied on significant opioid administration due to their potent analgesia amid a favorable hemodynamic profile,^14-16^ yet formulating an anesthetic plan which incorporates alternative analgesic adjuncts may confer advantages while avoiding a number of opioid-related side effects in this susceptible population (see Figure 2).^15^ When employed during cardiac surgery, opioid-sparing techniques have demonstrated earlier extubation times and improved oxygenation.^17^ Conversely, high-dose opioid administration with fentanyl or morphine has been associated with respiratory depression, delayed recovery, increased mechanical ventilation requirements, prolonged intensive care unit stay, and cost burden.^14^ Unfortunately, both pain-related respiratory stenting and opioid-related respiratory depression can precipitate physiologic changes that lead to right-sided heart failure. Other adverse effects associated with opioid use include ileus, urinary retention, constipation, delirium, pruritus, nausea, and vomiting.^9,18^ Opioids and particularly ultra-short-acting remifentanil have been implicated in the development of opioid-induced hyperalgesia and subsequently increased risk of PPP.^15,19-21^ The transition from acute to chronic pain and PPP may further contribute to opioid physical dependence and addiction.^8^ On the contrary, there is emerging evidence that long-acting opioids may minimize PPP. Murphy and colleagues randomized cardiac surgical patients to receive either fentanyl or methadone intraoperatively. Patients in the methadone group reported better pain scores at 1 month following surgery.^22^ The mechanism for this has been attributed to the N-methyl-D-aspartate (NMDA) receptor blocking activity of methadone, which has been associated with the prevention of opioid-induced hyperalgesia.^23^ A perioperative, opioid-sparing strategy utilizing analgesic alternatives and adjuncts may ultimately mitigate the risk of developing PPP and additional opioid-induced side effects,^15^ while also conferring additional benefits versus opioids alone.^15,24^ It is important to clarify, however, that prevailing evidence and expert opinion support opioid-sparing but not opioid-free techniques.^25^ Not only do opioids, when dosed in moderation, have longstanding efficacy, the absolute avoidance of them has actually been shown to cause harm.^26^

The application of multimodal analgesics may play particular importance in preventing PPP as this more inclusive approach has been described as providing optimal acute pain management with potentially fewer side effects when compared with a traditional opioid-based strategy.^9,15,16,24^ Acetaminophen, gabapentinoids, and ketamine have all been previously described as providing both effective analgesia and offering opioid-sparing benefit.^27,28^ Rafiq and colleagues conducted a prospective randomized controlled trial (RCT) examining multimodal analgesics versus opioids following cardiac surgery through median sternotomy.^24^ The authors found a combined regimen utilizing dexamethasone, gabapentin, ibuprofen, and acetaminophen provided superior analgesia (decreased numeric rating pain scores) through postoperative day 3 and decreased incidence of nausea and vomiting versus morphine and acetaminophen alone.^24^ Despite these findings during the acute pain period, a recent systematic review and meta-analysis regarding gabapentinoids observed no clinically significant analgesic effect to support their routine perioperative use.^29^ Further literature remains limited or not yet assessed regarding gabapentinoid use in the prevention of chronic poststernotomy pain.^27,30^

A subsequent systematic review conducted by Bignami and colleagues examined perioperative pain management modalities and cardiac surgery including multimodal analgesics.^16^ With regard to acetaminophen (paracetamol) administration, the authors found 2 of 4 RCTs demonstrating significantly decreased Visual Analogue Scale (VAS) scores and one of these publications demonstrating significantly decreased opioid consumption.^16,31-34^ Of 3 RCTs examining nonsteroidal anti-inflammatory drug (NSAID) analgesics and cardiac surgery, 2 trials demonstrated decreased VAS, and opioid consumption with NSAID use.^16,17,35,36^ Although the authors acknowledge the literature remains sparse regarding acetaminophen and NSAID use specific to cardiac surgery, Bignami and colleagues^16^ conclude that the use of multimodal analgesia provides synergistic pain relief with fewer adverse effects. Although VAS scores permit readily obtainable data gathering, more comprehensive assessments including functional and disability data may provide more reliable and objective measures in assessing pain.^37^ Direct comparisons of differing pain-related studies are often complicated by heterogeneity in outcome assessment and the use of non-patient-centric endpoints such as morphine equivalents. There are numerous validated patient satisfaction and quality of life questionnaires that better capture more meaningful outcomes related to improved pain control rather than relying on VAS scores alone.^38,39^

Beyond the acute postoperative period, the role of systemic pharmacologic therapies in preventing the development of chronic pain after surgery has also been examined. Chaparro and colleagues conducted a meta-analysis of RCTs with systemic drugs administered perioperatively and evaluated pain 3 or more months following surgery.^40^ Among the 40 RCTs with analgesics including NSAIDs, gabapentin, pregabalin, lidocaine, steroids, ketamine, NMDA blockers, fentanyl, nitrous oxide, venlafaxine, and the antiarrhythmic mexiletine, only intravenous ketamine provided a statistically significant, albeit modest, reduction in the incidence of postsurgical chronic pain.^40^ In a subsequent review of postoperative chronic pain prevention, Reddi^41^ similarly concluded that ketamine may reduce chronic pain after surgery while suggesting gabapentin and pregabalin as promising but requiring additional robust studies prior to recommendation. In a recent review of chronic poststernotomy pain, Kleiman and colleagues^27^ similarly found no evidence to support the use of transdermal lidocaine, glucocorticoids, or dexmedetomidine in decreasing chronic pain. In contrast, the authors acknowledge the effectiveness of acetaminophen, gabapentinoids, and ketamine in providing opioid-sparing analgesia, but describe their utility in preventing chronic poststernotomypain as limited or not yet examined.^27^

The application of regional anesthesia provides a diverse array of potentially opioid-sparing pain management methods in accordance with a multimodal analgesic strategy. The ability to administer local anesthetics through regional anesthesia may be particularly favorable in high-risk surgical candidates as these techniques may reduce or outright avoid adverse effects associated with opioid administration.^42^ Multimodal analgesics have often been combined with targeted regional anesthetic techniques to reduce opioid requirements, enhance pain control, and potentially reduce systemic analgesics and their accompanying side effects including delirium, hypotension, bradycardia, bleeding, and sedation.^43^ Neuraxial anesthesia serves as one regional technique that may be considered in cardiac surgery via thoracic epidural analgesia (TEA) or spinal anesthesia.^16,43,44^ TEA has been shown to provide effective analgesia with decreased VAS scoring and reduced opioid consumption postoperatively,^16,27^ yet no evidence supports a definitive decrease in developing chronic pain following TEA or intrathecal opioid administration.^27^ The risk of spinal hematoma has historically been shown to be 1:1528 for TEA and 1:3610 for spinal anesthesia,^45^ but the risk for TEA has more recently been shown to be as low as 1:3552.^46^ The 2018 guidelines released by the American Society of Regional Anesthesiologists states there is insufficient evidence to determine whether the risk of epidural hematoma with neuraxial procedures is increased with the use of the level of anticoagulation required for cardiopulmonary bypass (CPB).^47^ The decision to employ neuraxial anesthesia as part of any opioid-reducing analgesic strategy should always consider a risk-benefit calculation regarding epidural hematoma formation in the vulnerable cardiac surgery patient population.^9,16,43,48,49^

Thoracic paravertebral block (PVB) serves as alternative regional anesthetic technique utilized for analgesia related to cardiac surgery. Thoracic PVB consists of a type of peripheral nerve block with option for continuous catheter placement similar to TEA, yet PVB may be performed under direct ultrasound guidance. When compared with TEA, thoracic level PVB has been found to provide comparable analgesic efficacy and improved side effect profile, including theoretically less risk of epidural hematoma formation in the anticoagulated patient.^49-52^ The risk of epidural hematoma formation proves notably relevant within cardiac surgery due to the frequent anticoagulation requirement for patients undergoing cardiopulmonary bypass.^49^ Similar to TEA, there exists no evidence to support thoracic PVB in the prevention of chronic poststernotomy pain.^49^

Various other regional anesthetic techniques including emerging, ultrasound-guided peripheral nerve blocks have been applied for poststernotomy analgesia and cardiac surgery at large. Surgeon administered parasternal block has been shown to provide improved postoperative analgesia and reduced opioid requirements,^16,27^ without effect on chronic pain development.^27^ Similarly, ultrasound-guided parasternal intercostal nerve block performed presternotomy has demonstrated significantly lower postoperative pain scores versus saline injection^53^ or nonregional, morphine-based regimen.^54^ Studies assessing intrapleural block for sternotomy pain are limited, and similar to continuous wound catheters, have not yet examined their relationship with regard to developing PPP.^27^

Promising, emerging peripheral nerve blocks of the chest wall occur most often using direct visualization with ultrasound guidance, potentially adding to their safe application.^43,55^ The erector spinae plane (ESP) block presents a novel fascial plane block that was first described in 2016 for thoracic neuropathic pain^56^ and has since been applied as an easier to perform alternative to PVB to multiple procedure types involving the chest wall and abdomen.^49,57^ Few clinical studies assessing the ESP block for cardiac surgery have taken place, but initial data reveal promising analgesic efficacy and reduction in opioid consumption, while the need for replication via blinded RCTs persists.^49,58^ Similar analgesic studies have evaluated both the pectoral nerve and serratus anterior fascial plane blocks with encouraging results.^49^ The pectoralis (Pecs) nerve block entails dual interfascial plane injections (Pecs I and Pecs II) with the aim of anesthetizing the chest wall.^59^ Kumar and colleagues^60^ examined postoperative bilateral pectoralis nerve block following midline sternotomy, demonstrating both significantly decreased duration of ventilator dependence and improved pain scores utilizing the regional technique. Currently, no published evidence exists evaluating the more laterally performed serratus anterior nerve block approach specific to sternotomy pain, yet studies have shown improved pain scores following other thoracic surgical interventions.^61^

### A Team Approach

In addition to the consulting anesthesiologist’s role in managing opioid-sparing, multimodal analgesia, a multidisciplinary approach may provide the ideal strategy in preventing PPP in the cardiac surgery patient.^9,49,62^ Through incorporation of multiple individuals, a multidisciplinary, perioperative pain team may optimally treat complex pain through specialization, communication, and education among providers and patients. Such a team would be well positioned to combine clinical management with pain research.

Procedural considerations should be discussed with surgical colleagues as multiple operative variables may influence the development of PPP. For example, obtaining bilateral internal thoracic artery grafts via pedicled harvest rather than skeletonization has been associated with an increased risk of developing mediastinitis after coronary artery bypass graft (CABG).^27,63^ Because mediastinitis has been associated with developing chronic pain, the more selective skeletonization harvest may prove warranted, particularly in the diabetic patient population.^27^ Minimizing sternal retractor trauma, removing sternal wires posthealing, and prophylactic sternal plating in high-risk individuals has been suggested to minimize chronic pain contributions.^27^ Minimally invasive techniques have been used to decrease the degree of tissue traumatization, acute postoperative pain, intraoperative time, perioperative bleeding, and the incidence of sternal wound infections and recovery time.^64^ In addition, the removal of chest drainage tubes on the first postoperative day, as compared with days 2 or 3, has been associated with improved pain scores without an increased risk of requiring chest tube reinsertion.^13^

Surprisingly, it is unknown if the well-described state of heightened systemic inflammation associated with the use of CPB^65^ exacerbates acute postoperative pain. Despite the likelihood of a link between this altered pathophysiologic state and sensitization to pain signaling, this mechanism has not been investigated. There have been numerous studies comparing patients having undergone CABG surgery performed either with or without CPB, but none have specifically evaluated differences in pain following these techniques.^66^

There are some classes of home medications that may affect perioperative analgesia. To optimize analgesia while minimizing relapse and overdose, it is recommended for patients taking low-dose buprenorphine and methadone to continue those medications throughout the perioperative period.^67,68^ Interestingly, patients on cannabinoids may have higher tolerance to opioids and NSAIDs, requiring higher doses.^69^

Addressing patients’ psychological comorbidities may also play a key role within a multifaceted approach to PPP prevention after cardiac surgery. Patients may present with preexisting psychological risk factors for the development of chronic pain including anxiety, depression, posttraumatic stress disorder, and pain catastrophizing.^41,70-74^ Early involvement of a pain psychologist or integrative pain team may offer valuable insights to both identify and address these psychological risk factors outside of the cardiothoracic anesthesiologist and surgeon’s area of expertise.^71,72,75^

Many of the aforementioned pain management components and multidisciplinary approach relevant to cardiac-related PPP have been integrated as part of comprehensive, enhanced recovery after surgery (ERAS) protocols. ERAS pathways including those specific to cardiac surgery continue to gain popularity and aim to incorporate multiple objective variables to optimize patient experience, perioperative efficiency, and improve outcomes.^76-79^ Multimodal analgesia has remained central to ERAS protocols as this strategy may offer decreased opioid exposure in addition to potentially reducing recovery times, complications, and improving cost expenditures with surgery.^62,80,81^ Similarly, regional anesthesia has been increasingly implemented as part of a multimodal strategy in cardiac surgery and related ERAS protocols to reduce opioid consumption.^82,83^ The addition of emerging truncal fascial plane blocks for pain optimization presents a promising new area within cardiac ERAS.^55,84^ Because cardiac ERAS pathways often highlight opioid-sparing strategies and acute pain management optimization,^6,85,86^ implementation of these protocols aligns with addressing the aforementioned factors thought to contribute to PPP.

## Management of PPP

**Figure 3.**
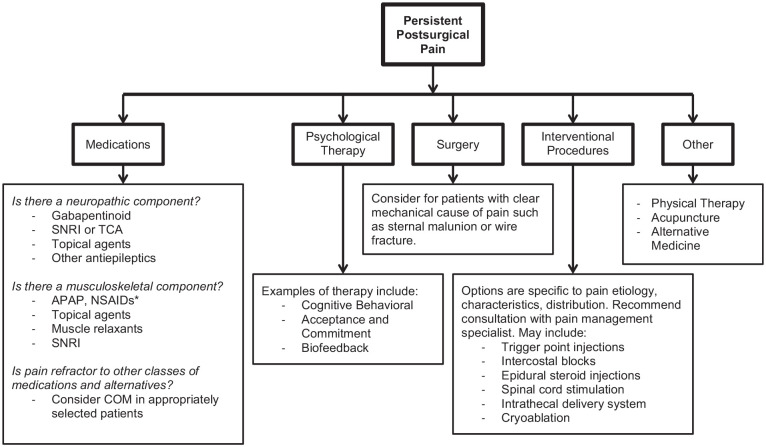
Treatment options available for persistent postsurgical pain.

Although prevention is likely the most impactful strategy to decrease the burden of PPP after cardiac surgery, this may not always be possible. There are a variety of treatment options for PPP including medications (see Figure 3 and Table 1), surgery, and interventional pain procedures.

**Table 1.** Medications to be Considered for Persistent Postsurgical Pain^a^.

Agents	Mechanisms	Comments
NSAIDs—ibuprofen, naproxen, and meloxicam	COX inhibition • Decrease peripheral production of inflammatory signaling molecules	Particularly effective during the acute phase of injury, although can be beneficial for chronic musculoskeletal pain. Chronic daily use caries increased risk of renal and cardiovascular complications.
Acetaminophen	COX inhibition • Central COX inhibition and other mechanisms	Separate mechanism for NSAIDs and can have a synergistic effect. Generally well tolerated when taken within recommended dose, but caution should be taken with hepatic impairment.
Gabapentinoids—pregabalin and gabapentin	Alpha2delta calcium channel inhibition • Decreased transmission of pain signals	First-line agents for treating neuropathic pain
SNRIs—duloxetine, venlafaxine, and milnacipran	Serotonin and norepinephrine reuptake inhibition • Modulation of descending inhibitory pathways	First-line agents for treating neuropathic pain, certain agents also have indication for other pain conditions including fibromyalgia and chronic musculoskeletal pain.
TCAs—nortriptyline, amitriptyline, and desipramine	Serotonin and norepinephrine reuptake inhibition. Also block histamine, sodium channels, and acetylcholine. • Modulation of descending inhibitory pathways	Well studied for neuropathic pain, in general, side effects can be more significant than SNRIs; however, dosing for pain is done at significantly lower doses than what is used for antidepressant effects.
Opioids—tramadol, oxycodone, hydrocodone, morphine, tapentadol, hydromorphone, methadone, and buprenoprhine	Opioid receptor activation • Central and peripheral analgesia	Considered for patients who have failed alternative medications and therapies and are appropriate candidates. Some opioids such as tramadol and tapentadol, which have SNRI properties, act at multiple sites on the pain pathway.

Abbreviations: NSAID, nonsteroidal anti-inflammatory; APAP, acetaminophen; SNRI, serotonin and norepinephrine reuptake inhibitor; TCA, tricyclic antidepressant.

a This table is not exhaustive but serves to highlight common medication classes used to treat postsurgical pain and examples of drugs in each class. In general, the type of pain that predominates for the patient should be considered. For example, a patient with predominantly burning, neuropathic pain following thoracotomy may have little effect from an NSAID and APAP, but respond favorably to a gabapentinoid and SNRI.

Medical management of PPP involves targeting the various pathways and mechanisms of pain. NSAIDs decrease the production of inflammatory cytokines and chemokines such as prostaglandins. This may be particularly helpful in the acute phase of injury for patients, but also plays a role in chronic musculoskeletal pain and associated flares. Both the acute and long-term risks of NSAID use needs to be considered, especially in the cardiac surgery population, and the distinction between acetylsalicylic acid (ASA) and other NSAIDs is warranted. Renal injury, increased risk of myocardial infarction and stroke are all potential adverse events that the cardiac surgery population may already have a higher baseline risk for developing when using non-ASA NSAIDs.^87,88^ Indeed, the most updated 2015 Food and Drug Administration (FDA) black box warning for non-ASA NSAIDs advises against their use in patients prior to or after CABG, emphasizing the potentially increased risk for heart attack or stroke in patients with underlying cardiovascular disease. Furthermore, the use of non-ASA NSAIDs for cardiac surgical patients has been associated with acute kidney injury,^89^ whereas ASA use has been shown to be protective.^90^

Acetaminophen or paracetamol is a known cyclooxygenase (COX) inhibitor, although it does not exert its effect through peripheral inhibition of this enzyme as the non-steroid anti-inflammatory class of medications.^91^ There is evidence that it inhibits COX centrally and acts through other non-COX mechanisms as well. This agent has a better safety profile without a demonstrated association with hepatic dysfunction, although caution should be taken in patients with acute liver injury.^92^

Gabapentin and pregabalin bind to the alpha2delta subunit of calcium channels and are used in treating many different types of chronic pain. They are most well studied and effective for neuropathic pain. For cardiac surgery patients that have a neuropathic component to their pain, these may be a reasonable treatment option and studies have shown that approximately half of cardiac surgical patients with PPP do have a neuropathic component to their pain.^1,5^ These agents have a generally favorable safety profile with nausea, sedation, and dizziness being some of the more common side effects and caution should be taken for patients with baseline renal dysfunction.

Additional neuropathic agents including serotonin and norepinephrine reuptake inhibitors (SNRIs) and tricyclic antidepressants (TCAs) may also play a role in the management of PPP that has a significant neuropathic component.^93^ The primary site of action is likely augmentation of the descending inhibitory pathways, although there are likely additional mechanisms of action that provide analgesic benefit.^94^ SNRIs including duloxetine have been studied for a variety of neuropathic pain conditions and shown to provide significant benefit.^95^ For most patients, SNRIs are better tolerated and have a better side-effect profile when compared with TCAs.

There is limited evidence regarding the use of dexamethasone specifically regarding the treatment postoperative pain in cardiac surgery patients. One randomized double blinded, placebo-controlled study of 300 postoperative patients who had coronary revascularization surgery did not find a difference in opioid consumption or severity of postoperative pain for the first 2 days after surgery.^96^

Chronic application of opioids needs to be carefully weighed with the risks. Chronic use needs to be carefully monitored and the appropriateness of ongoing therapy evaluated by pain management specialists.^97^ Patients should have treatment goals that may include pain reduction and functional improvement and they should respond favorably to low doses of opioids. Practically, the number of patients who should require and would benefit from chronic opioid therapy is likely low, although this has not been studied in this population.

Beyond medical management, interventional pain procedures may be appropriate for certain patients. Patients who have had a minimally invasive procedure performed via thoracotomy may develop intercostal neuralgia or post-thoracotomy pain. Treatment with intercostal nerve blocks, paravertebral blocks, trigger point injections, or thoracic epidural steroid injections may be treatment options.^98,99^ Cryoablation of neural tissue has been utilized for treatment of various painful conditions. The first study of cryoablation of the intercostal nerves was done in 1980 for pain associated with thoracotomy and reported significantly reduced analgesic use in the group that received the intercostal cryoablation.^100^ Perhaps the most relevant study pertinent to this review was done in 2000 for patients undergoing minimally invasive cardiac surgery via minithoracotomy.^101^ This study reported reduced pain scores, although no difference was seen in analgesic consumption. Neuromodulation is a field that has continued to develop and there are case reports and small studies of patients who may benefit for spinal cord stimulation for chronic, refractory angina or in one case truncal complex regional pain syndrome following sternotomy.^102,103^ A limitation of these techniques may be anticoagulation for patients with cardiovascular disease and further research is needed to determine which patients would benefit most from these advanced and more invasive techniques. Surgery can also be an option for a small subset of patients who have a clear mechanical component to their pain. This would include patients with sternal malunion or sternal wire fracture, although these are relatively rare complications and are likely not significant contributors to PPP for most patients.^104^

For refractory neuropathic pain antiepileptic agents (carbamazepine, lamotrigine, topiramate, etc), NMDA receptor antagonists such as memantine and infusions including ketamine or lidocaine are treatment options to consider.^93^ Treatment of chronic pain is an off-label use for most of these therapies given the relatively limited evidence, but for patients with refractory pain they may be appropriate to trial. Depending on the characteristics and location of the patient’s pain, topical agents such as lidocaine, diclofenac, or capsaicin can be considered. In patients with muscle spasms or myofascial pain, muscle relaxants (baclofen, tizanidine, methocarbamol, cyclobenzaprine, metaxalone, etc) can provide some symptom relief. Physical therapy is important for recovery in strength and function after surgery, but there can be further benefits for patients who go on to develop chronic pain. Dry needling can target pain with a myofascial component and therapists can work to improve function in patients who are deconditioned due to inactivity from their pain. Psychological therapy in the form of cognitive behavioral therapy, cognitive function therapy, acceptance and commitment therapy, biofeedback, and mindfulness can be helpful for patients with chronic pain, particularly when there are complicating psychosocial factors.^105^ The importance of a comprehensive approach to the treatment of chronic pain with therapy (physical and psychological), medication, interventions, and adjunctive therapies cannot be understated.

## Future Directions

PPP and opioid use remain vital pain management concerns in providing optimal anesthetic care surrounding cardiac surgery. Further best practices data are required through future research study in multiple areas including the following:

- Further studies identifying patient risk factors for the development of PPP after cardiac surgery
- Research needed to assess the effect of cardiac surgical procedure type and intraoperative interventions on pain outcomes
- Research examining the inflammatory state and role of intraoperative CPB use in the development of chronic pain
- Research addressing the consequence of acute pain management and its effect on the development of PPP
- Large-scale RCTs assessing systemic, multimodal analgesics in the development of chronic pain following cardiac surgery^40^
- Large-scale RCTs evaluating regional anesthesia applied to cardiac surgery and the development of chronic pain^16^
- Head-to-head comparative studies assessing novel, ultrasound-guided regional anesthetic techniques with cardiac surgery^49^
- Research assessing secondary hyperalgesia therapies as potential preventative measures to developing chronic pain^27^
- Research needed to assess the effect of psychological and behavioral interventions on the development of chronic postsurgical pain^72^
- Large-scale RCTs assessing ERAS pathway implementation and the development of chronic pain^49^
- Large-scale RCTs evaluating neuromodulation and spinal cord stimulator therapy for chronic poststernotomy pain

## Conclusion

Persistent postoperative pain after cardiac surgery represents a significant complication negatively affecting patients and health care systems. Adequately treating acute pain following cardiac surgery remains paramount as severe postoperative pain and opioid-induced hyperalgesia may increase the risk of developing PPP.^8,9,106^ Current literature suggests both prevention and treatment of PPP should optimally include multimodal pharmacology, regional anesthesia, and multidisciplinary involvement.^9,15,16,24,49,62^ Future research is required in this vulnerable patient population to confirm best practices in PPP prevention and treatment as part of an enhanced recovery after cardiac surgery pathway.
